# Inducible T Cell Costimulator Ligand and Inducible T Cell Costimulator Stratification Identify Dichotomous Tumor Microenvironment and Guide Chemo‐Immunotherapy in Small Cell Lung Cancer

**DOI:** 10.1002/mco2.70782

**Published:** 2026-05-28

**Authors:** Qiji Guo, Yan Chen, Jijun Sun, Hongyi Zhang, Shuyu Ji, Huansha Yu, Lele Zhang, Haiyang Hu, Peng Zhang, Jing Zhang

**Affiliations:** ^1^ Department of Thoracic Surgery Shanghai Pulmonary Hospital School of Medicine Tongji University Shanghai China; ^2^ Central Laboratory Innovation and Incubation Center Shanghai Pulmonary Hospital School of Medicine Tongji University Shanghai China

**Keywords:** biomarkers, immunotherapy, immunohistochemistry, small cell lung cancer, translational medical research

## Abstract

Inducible T cell costimulator ligand (ICOSLG) regulates T cell functional states, yet its role in small cell lung cancer (SCLC) remains poorly characterized. By integrating multiple cohorts, we found that high tumor‐intrinsic ICOSLG was associated with poor overall survival (OS) in the TU‐SCLC cohort (*p* = 0.010) and the Wang et al. cohort (*p* < 0.001), and was associated with inferior efficacy to chemo‐immunotherapy (hazard ratio [HR] = 2.66, *p* = 0.008). Consistently, the ICOSLG high subgroup exhibited significantly reduced functional CD8^+^ T cell infiltration, elevated exhausted CD8^+^ T cells, decreased effector molecules such as GZMK and IFNG, and enrichment of malignant pathways, including hypoxia, epithelial–mesenchymal transition, and others. Meanwhile, the correlation between ICOSLG and NEUROD1 expression was observed. On the contrary the subgroup high in ICOS, the main ICOSLG receptor, harbored NOTCH pathway mutations, showed an inflamed tumor microenvironment, better prognosis, and prolonged OS with chemo‐immunotherapy (HR = 0.52, *p* = 0.003). Dual ICOSLG and ICOS stratification revealed that the ICOSLG low and ICOS high subgroup derived the greatest benefit from chemo‐immunotherapy (median OS: 17.2 vs. 9.8 months; *p* < 0.001). Collectively, this dual‐stratification strategy refined patient selection for chemo‐immunotherapy, unveiled actionable targets, and ultimately advanced precision immunotherapy in SCLC.

## Graphical abstract

In small cell lung cancer, high tumor ICOSLG expression drives a microenvironment enriched with immunosuppressive T cells and M2 macrophages. Conversely, ICOSLG low tumors promote CD8^+^ T cell infiltration. Clinically, high ICOSLG levels predict poor response to immune checkpoint inhibitors and worse survival, whereas low ICOSLG expression indicates better clinical outcomes.

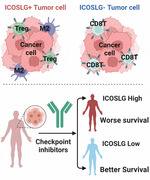

## 1 Introduction

Small cell lung cancer (SCLC) accounts for approximately 11% of all diagnosed lung cancers and leads to 250,000 deaths worldwide annually [1, 2]. Despite the continuous emergence of new therapies, the prognosis of SCLC remains unfavorable, with a median survival of less than 1 year [3]. Fortunately, immune checkpoint inhibitors (ICIs) shed light on the treatment landscape of SCLC. For example, adding atezolizumab, a programmed cell death ligand 1 (PD‐L1) antibody, to standard chemotherapy significantly prolonged the median overall survival (OS) in patients with extensive‐stage SCLC (ES‐SCLC) by 2 months [4]. Currently, several ICIs, including atezolizumab, durvalumab, serplulimab, and adebrelimab, have been approved for ES‐SCLC treatment [4, 5, 6, 7]. These breakthroughs have firmly established immunotherapy as a pivotal cornerstone in the modern management of ES‐SCLC.

Nevertheless, only a subset of patients achieves a striking response to ICIs, and some experience severe adverse events, driving investigations in pursuit of predictive biomarkers. Specifically, molecular classification using RNA sequencing had identified four distinct groups, most notably an inflamed (SCLC‐I) subtype. This subtyping demonstrated notable sensitivity to ICIs due to their immune‐infiltrated microenvironment. In contrast, the remaining subtypes were generally immunosuppressive and less responsive. However, a fraction of patients with other subtypes still derive clinical benefit, indicating that this classification might not fully encapsulate all potential responders. Furthermore, while molecular subtyping based on large‐scale sequencing has enabled the delineation of tailored treatment strategies, it poses significant challenges for practical application in routine clinical settings.

Inducible T cell costimulator ligand (ICOSLG), also known as B7‐H2, is a member of the B7 family of immune regulatory proteins. ICOSLG was typically expressed by antigen‐presenting cells (APCs), including macrophages, B cells, and monocytes [8]. Moreover, expression of ICOSLG had also been detected on tumor cells in various cancers, such as glioblastoma [9]. Through binding to its receptor ICOS on T cells, ICOSLG could stimulate T cell activation and antitumor immunity. Studies demonstrated dual roles of the ICOSLG–ICOS axis. In breast and nasopharyngeal carcinomas, chemotherapy‐induced ICOSLG on B cells enhanced cytotoxic T cell responses, correlating with tumor suppression and prolonged survival [10, 11]. Conversely, in hematologic malignancies and glioblastoma, ICOSLG expanded immunosuppressive Tregs or IL10^+^ T cells, thereby impacting immunotherapy efficacy [9, 12]. Notably, ICOSLG triggered by secreted phosphoprotein 1 (SPP1)‐encoded osteopontin induced cell migration, increased angiogenesis, and tumor metastasis [13]. ICOSLG playes a dual role in mediating the tumor microenvironment (TME), and little is known about the role of ICOSLG in SCLC.

In this study, we aimed to investigate the clinical role of ICOSLG and ICOS in predicting prognosis and immunotherapy response, both individually and synergistically. Furthermore, we performed a multiomics integrative analysis spanning transcriptomic, proteomic, and genomic landscapes to elucidate the impact of ICOSLG and ICOS on the TME, their underlying genomic signatures, and upstream regulatory mechanisms underpinning their prognostic and immunotherapeutic relevance. Overall, our findings provided a significant improvement over existing biomarkers and laid the foundation for precisely predicting immunotherapeutic efficacy.

## 2 Results

### 2.1 ICOSLG was Correlated With Adverse Prognosis and Efficacy of Chemo‐Immunotherapy in SCLC

**FIGURE 1:**
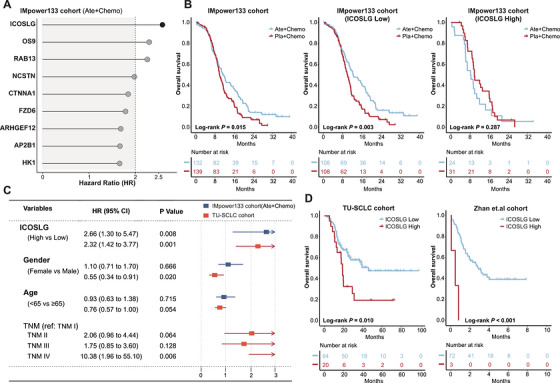
**ICOSLG overexpression was associated with adverse clinical outcomes and chemo‐immunotherapy efficacy in SCLC**. (A) The ten genes with the highest HR for chemo‐immunotherapy efficacy in the IMpower133 cohort. (B) Kaplan–Meier analysis of OS comparing patients treated with chemo‐immunotherapy versus chemotherapy alone in the overall population, ICOSLG low subgroup, and ICOSLG high subgroup in IMpower133 cohort. (C) Forest plot of multivariate Cox proportional hazards regression analysis including ICOSLG, gender, age, and TNM stage. (D) Kaplan–Meier analysis of OS between ICOSLG low subgroup and ICOSLG high subgroup in the TU‐SCLC cohort and Wang et al. cohort. A log‐rank test was conducted for Kaplan–Meier analysis.

To identify factors associated with prognosis and efficacy of chemo‐immunotherapy in SCLC, the hazard ratio (HR) of OS was calculated for all the genes in the IMpower133 cohort and the TU‐SCLC cohort (Figure S1A) [14, 15, 16]. We listed genes with HR > 1 in the IMpower133 cohort, genes with HR > 1 in the TU‐SCLC cohort, and the finally selected genes in Table S1. Among these genes, ICOSLG showed the highest HR (Figure 1A). After selecting ICOSLG as the candidate protein, we tested cut‐offs of 10, 20, 25, and 50% and found that all of them identified patients whose OS with chemo‐immunotherapy was similar to chemotherapy alone (Figure S1B–D). In clinical settings, an *H*‐score ≥ 200 is generally regarded as strong positivity and is easily recognized on tissue sections [17]. Because this threshold also corresponded to 20% of tumor cells expressing ICOSLG in our cohort, we adopted a 20% cut‐off for all subsequent analyses. Given the strong correlation between messenger RNA (mRNA) and ICOSLG protein (Figure S1E), the ICOSLG high group was defined as patients in the top 20% of mRNA level for the IMpower133 cohort without proteomic data. Unless otherwise specified, “ICOSLG high” referred to the top 20% of ICOSLG expression or protein levels. We first analyzed the clinicopathological characteristics (Table 1). In the limited‐stage TU‐SCLC cohort, the ICOSLG high subgroup exhibited significantly lower proportions of male patients and smokers, while age distribution remained balanced. Conversely, in the extensive‐stage IMpower133 cohort, ICOSLG status showed no significant correlation with clinical factors such as age or gender.

**TABLE 1:** Clinicopathological characteristics and relationships with ICOSLG status in TU‐SCLC and IMpower133 cohort.

Characteristics	TU‐SCLC cohort (*n* = 112)			IMpower133 cohort (*n* = 271)		
	ICOSLG status			ICOSLG status		
	High	Low	*p*	High	Low	*p*
Total	22	90		55	216	
Gender			0.002*			0.302
Male	15	83		38	133	
Female	7	7		17	83	
Smoking			0.016*			0.737
Current/previous	13	76		53	210	
Never	5	5		2	6	
Unknown	4	9		0	0	
Age			0.943			0.098
<65	14	58		25	125	
≥65	8	32		30	91	
Treatment			/			0.399
Atezolizumab	/	/		24	108	
Placebo	/	/		31	108	
ECOG			/			0.246
0	/	/		24	76	
1	/	/		31	140	
VALG stage			/			/
ES	0	0		55	216	
LS	22	90		0	0	
MLiver			/			0.640
No	/	/		35	130	
Yes	/	/		20	86	
MBrain			/			0.258
No	/	/		48	199	
Yes	/	/		7	17	
LDH			/			0.546
≤ULN	/	/		20	95	
>ULN	/	/		33	116	

Abbreviations: ECOG: Eastern Cooperative Oncology Group; ES: extensive‐stage; LDH: lactate dehydrogenase; LS: limited‐stage; SCLC: small cell lung cancer; ULN: upper limit of normal; VALG: Veterans Administration Lung Group.

* *p* value was used from Pearson's chi‐square test and Fisher's exact test, significant *p* value ≤ 0.05 was shown in bold.

Kaplan–Meier analyses further elucidated ICOSLG's clinical role. In the IMpower133 cohort, high ICOSLG expression was correlated with worse progression‐free survival (PFS), although the impact on OS (*p* = 0.056) was not statistically significant (Figure S1F,G). To further confirm its role as an immune‐related predictive biomarker rather than merely a prognostic factor, the full cohort was employed for subsequent ICOSLG high/low stratification analyses of OS and PFS. It turned out that the ICOSLG low subgroup treated with atezolizumab plus chemotherapy had better OS (*p* = 0.003) than those receiving chemotherapy alone, whereas no OS benefit was observed in the ICOSLG‐high subgroup (*p* = 0.287; Figure 1B). A similar trend was also observed for PFS (Figure S1H). Next, multivariate analysis revealed that ICOSLG stratification was the most significant predictor of prognosis in the TU‐SCLC cohort and of chemo‐immunotherapy efficacy in the IMpower133 cohort (Figure 1C). Meanwhile, this prognostic association was further validated in the Wang et al. cohort [18] (Figure 1D). Taken together, our data illustrated that ICOSLG stratification could serve as an independent prognostic factor and a predictor of chemo‐immunotherapy efficacy in SCLC.

### 2.2 ICOSLG Promoted Immune Evasion in SCLC by Orchestrating the TME

**FIGURE 2:**
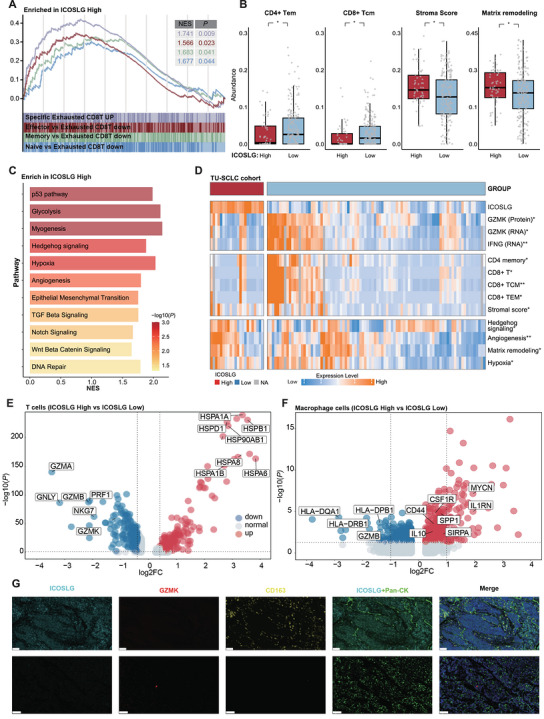
**ICOSLG‐mediated immune evasion in SCLC via tumor microenvironment remodeling**. (A) GSEA analysis evaluating the functional status of CD8^+^ T cells between ICOSLG high and ICOSLG low subgroups in the IMpower133 cohort. (B) Evaluation of CD4^+^ Tem, CD8^+^ Tcm, stromal score, and matrix‐remodeling score between ICOSLG high and ICOSLG low subgroups in the IMpower133 cohort. Statistical significance was determined by the Mann–Whitney *U* test. (C) Assessment of hallmark pathways between ICOSLG high and ICOSLG low subgroups in the IMpower133 cohort by GSEA. (D) Abundance of effector molecules, immune cells, and functional pathways for each patient classified as ICOSLG high or low in the TU‐SCLC cohort. Bars at the top of the heatmap indicated the definition of ICOSLG high or low. Data were analyzed by the Mann–Whitney *U* test. (E and F) Volcano of differentially expressed genes comparing T cells and macrophages derived from ICOSLG high or low patients by scRNA‐seq data. (G) Multiplex immunofluorescence staining showing the expression of ICOSLG, Pan‐CK, CD163, and GZMK in specimens of SCLC. Scale bars, 60 µm. *, *p* < 0.05; **, *p* < 0.01; ***, *p* < 0.001.

TME represents a pivotal component of cancer, and inflammation is a crucial component of TME [19, 20]. Hence, we first investigated immunophenotypic differences between ICOSLG subgroups in the IMpower133 cohort. Gene set enrichment analysis (GSEA) revealed that the naive, memory, and effector CD8^+^ T cells were downregulated compared with the exhausted CD8^+^ T cells (Figure 2A) [21]. Xcell algorithm analysis showed a lower level of CD4^+^ T effector memory (Tem), CD8^+^ T central memory (Tcm), along with higher stromal scores in the ICOSLG high subgroup (Figure 2B). The Matrix remodeling score evaluated by the single sample GSEA (ssGSEA) algorithm was also elevated (Figure 2B). We further assessed functional differences using hallmark gene signatures. By GSEA, abundant malignant pathways were enriched in the ICOSLG high subgroup, such as hedgehog signaling, hypoxia, epithelial–mesenchymal transition (EMT), and angiogenesis (Figure 2C). In the TU‐SCLC cohort, some discoveries were further reinforced, such as the enrichment of hedgehog signaling, hypoxia, angiogenesis, and matrix remodeling, the downregulation of CD4^+^ T memory and CD8^+^ Tcm (Figure 2D). To further characterize the TME, public single‐cell RNA sequencing (scRNA‐seq) data were employed to compare the functional status of T cells and macrophages between two subgroups (Figure S1I). In the ICOSLG high subgroup, effector molecules such as granzyme K (GZMK), granzyme A (GZMA), and interferon‐gamma (IFNG) were highly downregulated, and heat shock proteins (HSPs) were enriched in T cells (Figure 2E). For macrophages, SPP1, interleukin 1 receptor antagonist, and signal regulatory protein alpha were enriched in ICOSLG high subgroup and granzyme B (GZMB) and human leukocyte antigen (HLA) subtypes (HLA–DQA1/DRB1) were enriched in ICOSLG low subgroups (Figure 2F). Consistently, immunofluorescence on SCLC samples revealed that tumors with high ICOSLG expression contained significantly more CD163^+^ cells, which represented M2 macrophages, and fewer GZMK^+^ cells, which represented cytotoxic T lymphocytes, than ICOSLG low tumors (Figure 2G). Furthermore, we conducted an in vitro functional validation experiment using an ICOSLG neutralizing antibody. Blocking ICOSLG resulted in a statistically significant decrease in the expression of the exhaustion marker EOMES and a significant increase in the effector molecule GZMA in the T cells (Figure S1J).

Collectively, these results indicated that high ICOSLG expression was associated with impaired T cell antitumor capacity and immunosuppressive macrophage function.

### 2.3 Tumor‐Intrinsic ICOSLG Levels Were Positively Associated With NEUROD1 Expression in SCLC Patients

**FIGURE 3:**
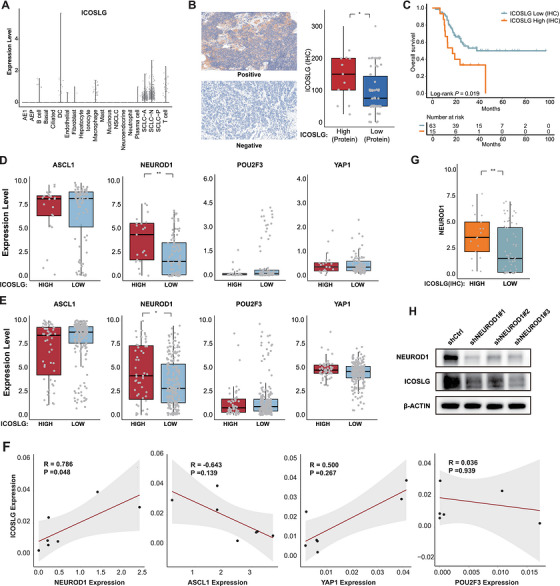
**NEUROD1 correlated with tumor‐intrinsic ICOSLG expression in SCLC**. (A) Quantitative analysis of ICOSLG expression levels across all cells in SCLC by scRNA‐seq data from the Rudin et al. cohort. (B) Representative histopathologic examples of ICOSLG expression and the IHC level between the ICOSLG high and ICOSLG low subgroups. (C) Kaplan–Meier analysis of OS between ICOSLG low subgroup and ICOSLG high subgroup classified by IHC level in TU‐SCLC cohort. (D and E) The mRNA expression profiles of transcription factors (ASCL1, NEUROD1, YAP1, POU2F3) between ICOSLG high and ICOSLG low subgroups in the IMpower133 cohort and TU‐SCLC cohort. Statistical significance was determined by the Mann–Whitney *U* test. (F) The Spearman correlation coefficient between the average expression of transcription factors and ICOSLG across all tumor cells per patient. (G) The expression of NEUROD1 between ICOSLG (IHC) high and ICOSLG (IHC) low subgroups. (H) Immunoblots showing the protein levels of the indicated proteins in H82 cell lines. shNEUROD1 indicated knockdown of NEUROD1. *, *p* < 0.05; **, *p* < 0.01; ***, *p* < 0.001.

ICOSLG was primarily expressed by APCs but had also been reported in solid tumors such as glioma [8, 9]. Using public scRNA‐seq data, we identified SCLC cells as the primary source of ICOSLG, particularly in the SCLC‐N subtype (Figure 3A) [22]. Immunohistochemistry (IHC) further confirmed that SCLC cells exhibited significant ICOSLG expression, which correlated with poor prognosis (Figure 3B,C).

Traditionally, four transcription factors, including Achaete‐scute family bHLH transcription factor 1 (ASCL1), neurogenic differentiation 1 (NEUROD1), POU class 2 homeobox 3 (POU2F3), and Yes1‐associated transcriptional regulator (YAP1), played a pivotal role in SCLC [23]. We analyzed mRNA expression of these transcription factors in ICOSLG high and low subgroups. In both the IMpower133 cohort and the TU‐SCLC cohort, the ICOSLG high subgroup showed elevated NEUROD1 compared with the ICOSLG low subgroup (Figure 3D,E). However, no significant differences were found in the expression of ASCL1, POU2F3, or YAP1 between subgroups. This positive correlation between ICOSLG and NEUROD1 was further validated in scRNA‐seq data and IHC level (Figure 3F,G). NEUROD1, as a transcription factor, regulates gene transcription by binding to specific DNA sequences. To interrogate the putative regulation of ICOSLG by NEUROD1 at the cellular level, shRNA‐mediated knockdown of NEUROD1 in H82 was performed and revealed a marked reduction in ICOSLG protein after knockdown of NEUROD1 (Figure 3H). To gain insight into the correlation, we analyzed public chromatin immunoprecipitation sequencing (ChIP‐seq) data from NEUROD1 high H524 cells and identified a peak annotated to ICOSLG via the GREAT algorithm (Figure S2A–C) [24, 25]. Meanwhile, we observed consistent positive correlations between NEUROD1 and ICOSLG expression in adrenocortical carcinoma and prostate adenocarcinoma (Figure S2D,E). Overall, these results indicated the potential correlation between NEUROD1 and ICOSLG in SCLC cells.

### 2.4 ICOS Expression Associated With an Inflamed Microenvironment and Indicated Survival Benefits Derived From Chemo‐Immunotherapy

**FIGURE 4:**
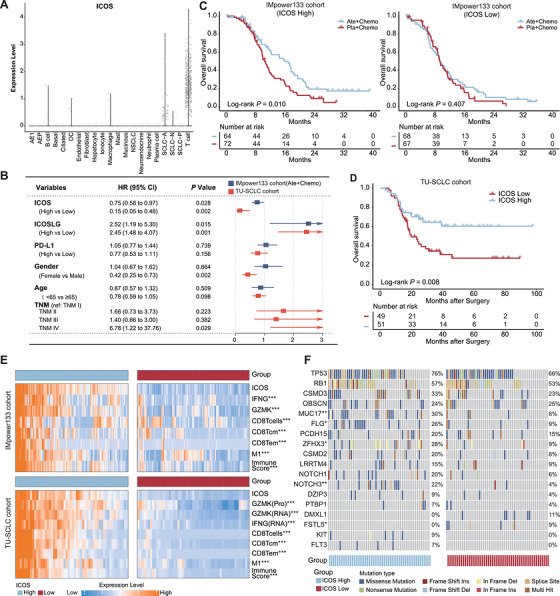
**ICOS enrichment predicted inflamed phenotypes and survival benefits in chemo‐immunotherapy treated SCLC**. (A) Quantitative analysis of ICOS expression levels across all cell types evaluated by scRNA‐seq data. (B) Forest plot of multivariate analyses including ICOS, ICOSLG, PD‐L1, gender, age, and TNM stage, conducted by cox proportional hazards regression model. (C) Kaplan–Meier analyses of OS among patients who have received chemo‐immunotherapy or chemotherapy alone in ICOS high and ICOS low subgroups. (D) Kaplan–Meier analysis of prognosis stratified by ICOS expression. (E) Abundance of effector molecules and immune cells for each patient showing ICOS high or low in the IMpower133 cohort and TU‐SCLC cohort. Bars at the top of the heatmap indicated the definition of ICOS high or low. Data were analyzed by the Mann–Whitney *U* test. (F) The waterfall plot of significantly mutated genes between ICOS high or low subgroups. *p* is derived from Pearson's chi‐squared test. Log‐rank test was conducted for Kaplan–Meier curves. *p* < 0.05 was considered statistically significant. *, *p* < 0.05; **, *p* < 0.01; ***, *p* < 0.001.

ICOS is considered the major receptor of ICOSLG and plays an indispensable role in immune responses [26, 27, 28, 29, 30]. To characterize the role of ICOS in SCLC, we analyzed scRNA‐seq data, revealing predominant ICOS expression in T cells (Figure 4A). We further examined the association between ICOS expression and clinical features (Table 2). In the TU‐SCLC cohort, the ICOS high subgroup was characterized by higher proportions of males and smokers. In the IMpower133 cohort, high ICOS expression was significantly associated with better performance status, lower incidence of liver metastasis, and lower lactate dehydrogenase (LDH) levels. Multivariate Cox regression analyses of OS were performed to assess clinical roles in two cohorts. Furthermore we found that higher expression of ICOS was associated with better survival benefit and superior efficacy of chemo‐immunotherapy, serving as a biomarker candidate independent of PD‐L1, ICOSLG (Figure 4B). Kaplan–Meier analysis further confirmed these findings. ICOS high subgroup had significantly longer OS after chemo‐immunotherapy compared with chemotherapy alone and also better prognosis compared with that in ICOS low subgroup (Figure 4C,D). Abundant infiltrated immune cells of CD8^+^ T cells, CD8^+^ Tcm, CD8^+^ Tem, macrophages, plentiful effector molecules, expression of GZMK, IFNG were discovered in the ICOS high subgroup in both IMpower133 cohort and TU‐SCLC cohort (Figure 4E). Next, whole exome sequencing data were interrogated to illuminate the genomic characteristics of ICOS high patients. Frequently, mutations of *mucin 17* (*MUC17*), *zinc finger homeobox 3* (*ZFHX3*), *neurogenic locus notch homolog 3* (*NOTCH3*), and *neurogenic locus notch homolog 1* (*NOTCH1*) were observed in the ICOS high subgroup (Figure 4F). Our prior study demonstrated that the *ZFHX3* mutation was significantly enriched in the immune‐inflamed subtype of SCLC and strongly correlated with increased immune cell infiltration [14]. Additionally, in the majority of SCLC cases, loss‐of‐function mutations in *NOTCH* genes and ectopic activation of the Notch signaling pathway both exhibited tumor‐suppressive effects [31]. After adding the immune‐score covariate, the correlations between *ZFHX3* mutations and ICOS were no longer significant, suggesting that their initial association was largely driven by overall immune infiltration (Figure S3A). In contrast, the correlations between *NOTCH3* mutations and ICOS remained significant. We extended this correction to all other genes, and the results were listed in Table S2. In general, these results implied that high ICOS expression possessed a unique mutational profile and was linked to an inflammatory TME, serving as an independent factor of prognosis and efficacy of chemo‐immunotherapy in SCLC.

**TABLE 2:** Clinicopathological characteristics and relationships with ICOS status in TU‐SCLC and IMpower133 cohort.

Characteristics	TU‐SCLC cohort (*n* = 112)			IMpower133 cohort (*n* = 271)		
	ICOS status			ICOS status		
	High	Low	*p*	High	Low	*p*
Total	54	53		136	135	
Gender			0.004*			0.766
Male	52	41		87	84	
Female	2	12		49	51	
Smoking			0.002*			0.148
Current/previous	50	35		134	129	
Never	1	9		2	6	
Unknown	3	9				
Age			0.499			0.946
<65	36	32		75	75	
≥65	18	21		61	60	
Treatment			/			0.586
Atezolizumab	/	/		64	68	
Placebo	/	/		72	67	
ECOG			/			0.049*
0	/	/		58	42	
1	/	/		78	93	
VALG stage			/			/
ES	0	0		55	216	
LS	54	53		0	0	
MLiver						0.041*
No	/	/		91	74	
Yes	/	/		45	61	
MBrain			/			0.084
No	/	/		128	119	
Yes	/	/		8	16	
LDH			/			0.005*
≤ULN	/	/		71	44	
>ULN	/	/		62	87	

Abbreviations: ECOG: Eastern Cooperative Oncology Group; ES: extensive‐stage; LDH: lactate dehydrogenase; LS: limited‐stage; SCLC: small cell lung cancer; ULN: upper limit of normal; VALG: Veterans Administration Lung Group.

* *p* value was used from Pearson's chi‐square test and Fisher's exact test, significant *p* value ≤ 0.05 was shown in bold.

### 2.5 A Combination of ICOSLG and ICOS Refined the Clinical Prediction of Response to Chemo‐Immunotherapy and Indicated Potential Vulnerability

**FIGURE 5:**
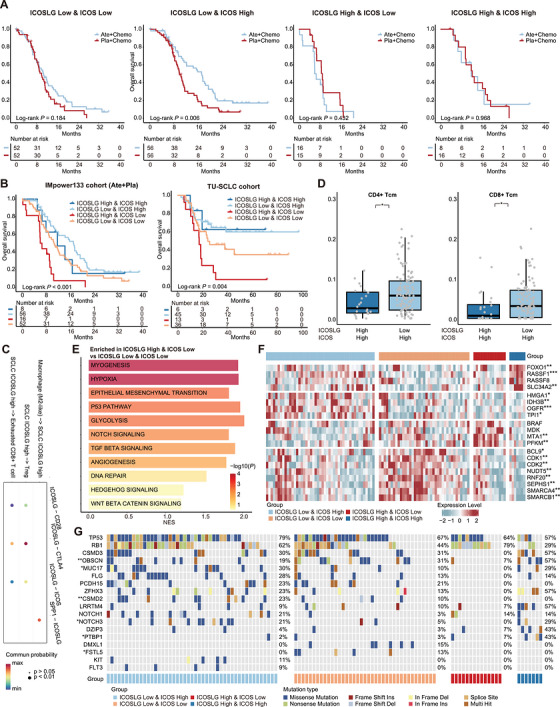
Dual ICOSLG/ICOS stratification refined chemo‐immunotherapy response prediction and revealed therapeutic vulnerabilities. (A) Kaplan–Meier analysis of OS among patients who have received chemo‐immunotherapy or chemotherapy alone in dual ICOSLG/ICOS stratification. (B) Kaplan–Meier analysis of OS among patients who have received chemo‐immunotherapy and prognosis in dual ICOSLG/ICOS stratification. (C) Cell–cell interactions between ICOSLG‐expressed tumor cells and immune cell clusters containing T cells and macrophages in the tumor microenvironment inferred by CellChat. (D) Abundance of CD4^+^ Tcm and CD8^+^ Tcm between high and low ICOSLG expression within high ICOS expression subgroups. (E) GSEA analysis of hallmark pathways between high and low ICOSLG expression within low ICOS expression subgroups. (F‐G) Genes specifically upregulated or mutated among the four groups. *, *p* < 0.05; **, *p* < 0.01; ***, *p* < 0.001.

Given the independent predictive value of ICOSLG and ICOS, we thus investigated their combined effect on predicting treatment outcomes. Among the four subgroups, only the ICOSLG low and ICOS high subgroups showed a significant benefit from chemo‐immunotherapy versus chemotherapy alone (Figure 5A). In both the IMpower133 and TU‐SCLC cohorts, the ICOSLG high and ICOS low subgroups presented the worst prognosis or efficacy of chemo‐immunotherapy (Figure 5B). Furthermore, we reconstructed Cox models for the IMpower133 cohort that include treatment, ICOS, ICOSLG, and their interaction while adjusting for age, gender, ECOG PS, liver or brain metastases, LDH, smoking status, SCLC transcriptional subtype, and PD‐L1 expression (Figure S3B). As all enrolled patients had ES‐SCLC in the IMpower133 cohort, TNM staging was not applicable. Accordingly, the interaction between ICOSLG and treatment remained significant (HR 2.65, 95% CI 1.10–6.42, *p* = 0.030) after full covariate adjustment, confirming that higher ICOSLG expression attenuated chemo‐immunotherapy benefit, whereas ICOS itself emerged as a strong positive prognostic factor. We also computed time‐dependent AUCs and discovered that the dual‐marker model outperformed the SCLC‐I classifier (Figure S3C). However, due to the lack of publicly available cohorts of limited‐stage SCLC (LS‐SCLC) patients receiving immunotherapy, we were unable to validate this in a large‐scale LS‐SCLC cohort. Nevertheless, we collected clinical samples from LS‐SCLC patients after immunotherapy and performed immunohistochemical staining for ICOS/ICOSLG (Figure S3D,E). The relevant results indicated that in patients with higher residual tumor after immunotherapy, tumor cells exhibited stronger ICOSLG expression intensity, while the level of infiltrating ICOS^+^ T cells was lower, providing preliminary validation for the predictive role of ICOSLG/ICOS in LS‐SCLC patients receiving immunotherapy.

To figure out the different TME of ICOSLG and ICOS subgroups, we annotated T cells and macrophages in SCLC and performed cell–cell communications by CellChat (Figure S4) [32]. Notably, tumor cells with ICOSLG expression interacted with exhausted CD8^+^ T cells, Tregs, and M2‐like macrophages via the ICOSLG pathway (Figure 5C). Specifically, the interaction between tumor cells and exhausted CD8^+^ T cells or Tregs through the ICOSLG−cytotoxic T‐lymphocyte associated protein 4 (CTLA4) axis was found to be more robust than that between ICOSLG and ICOS. Given the strong association between ICOS and the inflamed TME, we compared the differences between high and low ICOSLG expression stratified by ICOS expression. Within the ICOS high subgroup, the ICOSLG high subgroup demonstrated lower levels of CD4^+^ Tcm and CD8^+^ Tcm versus the ICOSLG low subgroup (Figure 5D). Whereas in the ICOS low subgroup, the ICOSLG high group exhibited more malignant tumor characteristics, such as hypoxia, EMT, glycolysis, and angiogenesis pathways, relative to the ICOSLG low subgroup (Figure 5E). Finally, integrated transcriptomic and genomic analyses identified subgroup‐specific potential therapeutic targets (Figure 5F,G). Specifically, the subgroup that benefited most from chemo‐immunotherapy, the ICOSLG low and ICOS high group, showed a significantly higher mutation frequency of *CUB and Sushi multiple domains 3* (*CSMD3*)*, MUC17*, and *NOTCH3*, indicating its potential genome background. The ICOSLG low and ICOS low subgroups exhibited elevated expression of cyclin‐dependent kinase 1 (CDK1) and cyclin‐dependent kinase 2 (CDK2) in the TU‐SCLC cohort (Figure S5). Notably, investigators had shown that CDK4/6 inhibitors combined with chemotherapy markedly suppressed SCLC growth in spontaneous mouse models. Recent studies highlighted that the multi‐CDK inhibitor dinaciclib showed potent antitumor activity in both human and mouse SCLC models [33]. Dinaciclib inhibited not only CDK9 but also CDK1, CDK2, and CDK5. The higher level of CDK1 and CDK2 in the ICOSLG low and ICOS low subgroups suggested the potential benefit of CDK‐related inhibitors. In conclusion, the combination of ICOSLG and ICOS refined clinical stratification and highlighted subgroup‐specific vulnerabilities for precision therapy in SCLC.

## 3 Discussion

Recently, the administration of ICIs has revolutionized cancer treatment, particularly in SCLC. The combination of immunotherapy and chemotherapy has become the standard treatment strategy for ES‐SCLC [4]. Despite this, nonimmune subtypes of SCLC often fail to derive durable benefits from ICIs, which necessitates exploration of mechanisms of treatment resistance [23]. Notably, the remarkable plasticity of SCLC tumor cells represents a critical contributor to therapeutic resistance in SCLC [22]. Therefore, accurately defining the pivotal subgroups of tumor cells and comprehending their functional mechanisms in immune regulation and clinical significance can significantly enhance the efficacy of ICIs in the treatment of SCLC.

Research has demonstrated that the B7 family plays a critical role in maintaining the balance between immune efficacy and suppression of autoimmunity [34]. The expression of B7 family members has been widely detected on numerous tumor cells, among which the most well‐known is B7H1, also known as PD‐L1 [35]. Specifically, B7 family members can interact with membrane proteins of T cells, such as PD1, ICOS, CD28, and CTLA4, thereby influencing the differentiation and functional states of T cells and subsequently modulating the TME. ICOSLG, also known as B7H2, was found to be expressed in tumor cells such as glioblastoma and could promote the formation of an immunosuppressive TME by expanding immunosuppressive IL10^+^ T cells [9]. In this study, we demonstrated that ICOSLG was also expressed in SCLC tumor cells. Consistently, we observed that the ICOSLG high subgroup exhibited enrichment of exhausted T cells and reduction in CD4^+^ Tem and CD8^+^ Tcm. While we did not observe elevated IL10 expression, scRNA‐seq analysis revealed upregulation of HSPs and downregulation of effector molecules in T cells. A previous study proved that T cells characterized by the expression of HSPs were significantly associated with immunotherapy resistance [36]. Additionally, the B7 family is regulated by various factors at the genomic, transcriptomic, posttranscriptional, and posttranslational level [37]. For instance, the expression of PD‐L1 was regulated by transcription factors, including MYC, IRF1, and HIF‐1α. In this study, ICOSLG was strongly correlated with NEUROD1, and an in vitro experiment showed ICOSLG might be regulated by NEUROD1. Collectively, these data suggested that ICOSLG^+^ tumor cells drove tumor immune evasion, potentially by impairing the function and reducing infiltration of T cells in SCLC.

Complex intercellular interactions are coordinated to influence the formation and evolution of the TME [38]. By recruiting and reprogramming immune cells, as well as remodeling the tumor vasculature and the extracellular matrix, tumor cells often shape a protumorigenic microenvironment. Characterizing intercellular communication networks within critical subgroups of tumor cells may reveal novel resistance mechanisms and uncover actionable combined therapy. Through cell communication analysis, we revealed that ICOSLG^+^ tumor cells exhibit a stronger interaction level with exhausted CD8^+^ T cells or Tregs via the ICOSLG–CTLA4 axis compared with that of the ICOSLG–ICOS or ICOSLG–CD28 interaction. Previous research had demonstrated that ICOSLG was also a ligand for CTLA4 and CD28 molecule [39]. As a critical immune checkpoint molecule primarily expressed on the surface of regulatory T cells and activated effector T cells, CTLA4 can suppress T cell‐mediated antitumor responses. The aforementioned results partially explained the different TME in ICOS or ICOSLG high subgroups. As the main receptor of ICOSLG, ICOS is primarily expressed on activated T cells and can induce cell proliferation, survival, and differentiation [40]. In our study, the ICOS high subgroup exhibited an inflamed immune phenotype, indicating that activation of ICOS enhanced the antitumor function of T cells and shaped the TME. Although elevated T cell infiltration was observed in patients with high ICOS expression, tumor cells in ICOSLG‐high patients may suppress T cell functionality through the ICOSLG–CTLA4 axis. Besides, we also noticed that tumor cells and macrophages could interact through the SPP1‐ICOSLG axis. Our previous research proved that SPP1^+^ macrophages were M2‐like polarized immune cells that drive immunosuppression, tumor progression, and fibrotic remodeling via the SPP1 axis [41]. Together, these results established ICOSLG^+^ tumor cells as a central regulator of TME in SCLC. ICOSLG blockade might be able to promote reprogramming of TME, thus potentially improve survival outcomes of patients with SCLC.

Precision medicine aims to accurately classify patients by using clinically actionable biomarkers. Despite the remarkable achievements of immunotherapy in SCLC, the lack of robust biomarkers still diminishes the therapeutic efficacy of immunotherapy. In our research, patients with low ICOSLG and high ICOS expression showed the superior sensitivity to chemo‐immunotherapy, achieving a median OS of 17.2 months with combination therapy versus 9.8 months with chemotherapy alone (*p* < 0.001). This survival benefit surpassed the improvement observed in transcriptomic subtype‐based selection: within the SCLC‐I subgroup (*n* = 49/271), combination therapy extended median OS from 10.4 to 18.2 months, yet our dual biomarker model identified a larger target population (*n* = 112/271) with comparable efficacy. This combinatorial stratification of ICOSLG and ICOS refined chemo‐immunotherapy response prediction and proposed potential clinically applicable biomarkers.

There are also some limitations to our study. To begin with, patients enrolled in the TU‐SCLC cohort had LS‐SCLC, which was distinct from ES‐SCLC in the IMpower133 cohort. Despite our findings that ICOS and ICOSLG were consistently implicated in prognostic prediction and exhibited promising predictive value for immunotherapy efficacy in ES‐SCLC, publicly available cohorts of LS‐SCLC patients treated with immunotherapy were lacking. Though we collected a small set of clinical specimens from LS‐SCLC patients after immunotherapy and performed ICOS/ICOSLG immunohistochemical staining as an initial verification, larger cohorts or prospective clinical trials are still required to validate these observations. Second, a positive correlation between ICOSLG and NEUROD1 was observed across the TU‐SCLC, IMpower133, and Rudin et al. cohorts.Although this correlation was supported by preliminary in vitro knockdown assays and in silico ChIP‐seq analysis, the direct transcriptional regulation mechanism remains to be fully elucidated. Future studies employing techniques such as ChIP‐qPCR or luciferase reporter assays are required to definitively validate the direct binding of NEUROD1 to the ICOSLG enhancer regions. Finally, given the retrospective design of our analysis, these results still require further validation within the framework of prospective, multicentered clinical cohorts.

To sum up, our study demonstrated the vital role of ICOSLG^+^ tumor cells in regulating TME and ultimately impairing survival benefit from ICIs in SCLC patients. Collectively, our current study not only manifested that ICOSLG and ICOS might serve as independent prognostic indicators, but also provided a roadmap for optimizing prediction of chemo‐immunotherapy efficacy by integrating ICOSLG and ICOS into a unified stratification model, which also highlighted ICOSLG as a promising therapeutic target for the treatment of SCLC.

## 4 Methods

### 4.1 Study Cohorts

Our study population included patient‐level data from four cohorts: transcriptomic and clinical data for chemo‐immunotherapy or chemotherapy alone from IMpower133 cohort (*n = *271) [42]; genomic, transcriptomic, proteomic, and clinical data for treatment naive patients from TU‐SCLC cohort (*n = *112) [14]; proteomic and clinical data for treatment naive patients from Wang et al. cohort (*n = *75) [18]; clinical data and scRNA‐seq data from Rudin et al. cohort (*n = *7) [23]. Notably, the TU‐SCLC cohort is derived from our prior work with data already publicly accessible, while the remaining cohorts were obtained from external public repositories.

### 4.2 Processing of Transcriptomic, Proteomic, and Genomic Data

For proteomic analyses, the protein matrix of the TU‐SCLC cohort and the Wang et al. cohort was obtained. The DreamAI ensemble algorithm (https://github.com/WangLab‐MSSM/DreamAI) was applied to impute missing values using the DreamAI R package. Only those proteins with a missing rate less than 50% were imputed. After imputation, 9559 proteins were used for downstream analyses. In the Wang et al. cohort, only three patients presented ICOSLG expression over zero, which was defined as the ICOSLG high subgroup.

For transcriptomic analyses, RNA sequencing data from the IMpower133 cohort and TU‐SCLC cohort were obtained as transcripts per million. The infiltration score of 64 types of immune cells, immune score, and stromal score were calculated by the Xcell algorithm [43]. The involved signatures for ssGSEA algorithms or GSEA were defined by previous studies (detailed in Table S3). Due to the absence of ICOS protein data in the TU‐SCLC cohort, ICOS subgroups were defined based on transcriptional levels in both the TU‐SCLC and IMpower133 cohorts. The median value, which was the most common method for initial biomarker exploration, was used as the cut‐off value for ICOS. Correlation analysis between NEUROD1 and ICOSLG expression levels in Adrenocortical Carcinoma and Prostate Adenocarcinoma was generated by TIMER [44, 45].

For genomic analyses, data were obtained from previously published research in the TU‐SCLC cohort. The mutation frequency of genes and the waterfall plot were made through the maftools package.

### 4.3 Processing of scRNA‐seq Data

To identify subpopulations, we further clustered T cells and macrophages. The gene expression matrices were normalized using the NormalizeData function with default parameters. The normalized data were then scaled using the ScaleData function. The top 2000 highly variable genes were detected using the FindVariableFeatures function. Principal component analysis was performed based on the 2000 most variable features using the RunPCA function. To adjust potential variation, we used Harmony (version 0.1.0) with default settings [46]. The first 20 principal components and a resolution of 0.2 were used with the FindNeighbors and FindClusters functions. Dimension reduction was performed using the RunUMAP function.

CellChat (version 1.1.3) [32] was utilized to infer ligand–receptor interactions. After annotating the object with relevant labels and identifying overexpressed genes, the communication probability was inferred using the computeCommunProb function. Cell–cell communications for each cell signaling pathway were generated with the computeCommunProbPathway function. Graphs were generated using the netVisual_chord_gene and netVisual_bubble functions.

### 4.4 IHC and Assay Methods

Serial tissue sections fixed in formalin and embedded in paraffin were used to construct tissue microarrays. Slides were stained with ICOSLG antibody (1:100; Abcam; ab257321) according to previous protocols [47]. The IHC score was calculated by the proportion of positive cells of tumor tissue (0–100%) by the average intensity of the positive staining (negative staining as 0, weak staining as 1, moderate staining as 2, and strong staining as 3) to obtain the score ranging from 0 to 300 for each sample. The immunohistochemical score of ICOSLG was evaluated under microscopy by two independent pathologists with relevant pathological reading abilities without knowledge of the patient's clinical information. The evaluations were performed simultaneously in the same reading room, thereby minimizing batch variability. The interobserver agreement was substantial (*κ* = 0.630, 95% CI: 0.526–0.734, *p* < 0.001), and the mean value derived from their respective evaluations was used as the basis for analysis.

### 4.5 Immunofluorescence and Assay Methods

Fluorescent double‐labeling was performed using species‐specific primary antibodies: ICOSLG mouse antihuman antibody (Abcam; ab257321; 1:200) and GZMK rabbit antihuman antibody (Servicebio; GB112190‐100; 1:500). After primary antibody incubation, horseradish peroxidase (HRP)‐conjugated secondary antibodies were applied, followed by tyramide signal amplification (TSA) to enhance detection sensitivity for low‐abundance targets. To prevent cross‐reactivity between sequential TSA cycles, microwave‐mediated HRP inactivation was rigorously performed after each tyramide reaction. Nuclei were counterstained with DAPI (Sigma–Aldrich; D9542), and autofluorescence quenching steps were integrated to minimize background interference. Finally, multispectral imaging of the immunolabeled slides was conducted using the 3DHISTECH Pannoramic MIDI slide scanner, capturing high‐resolution fluorescence signals across DAPI (nuclear), CY3 (red), and Alexa Fluor 488 (green) channels for colocalization analysis.

### 4.6 Cell Culture

The NCI‐H82 cell line (human SCLC, male), HEK‐293T cell line (human embryonic kidney), and Jurkat cell line (human lymphoblast, male) were obtained from American Type Culture Collection (ATCC). NCI‐H82 and Jurkat were cultured in RPMI‐1640 medium (Gibco; C11875500) and HEK‐293T in Dulbecco's modified Eagle medium (Gibco; C11995500), both supplemented with 10% fetal bovine serum (Sigma; F8318) and 1% penicillin/streptomycin (Gibco; 15140122). All cell lines used were cultured in 37°C humidified incubator with 5% CO_2_, and routinely confirmed as pathogen‐free by PCR.

### 4.7 Lentiviral Plasmid Construction and Lentiviral Transduction

Plasmid construction was carried out according to the manufacturer's instructions. DNA sequences encoding shRNAs were cloned into pLKO.1 plasmid (Addgene). The shRNA sequences used were as follows:

human shNEUROD1#1, 5′‐GCAACTCAATCCTCGGACTTT‐3′;
human shNEUROD1#2, 5′‐GCCTTGCTATTCTAAGACGCA‐3′;
human shNEUROD1#3, 5′‐GACAATATTATGTCCTTCGAT‐3′;

Lentiviral and packaging plasmids (pMD2.G and psPAX2; Addgene) were cotransfected into 293 T cells using Lipofectamine 2000 transfection reagent (ThermoFisher; 11668019). Lentiviral particles were collected 48 h after transfection and filtered with a 0.45 µm filter. Tumor cells were transduced and selected using puromycin (Gibco; A1113803). Knockdown efficiency was assessed by immunoblotting.

### 4.8 Quantitative Real‐Time PCR

For qRT‐PCR, total RNA was extracted from cells using FastPure Cell/Tissue Total RNA Isolation Kit (Vazyme; RC102), and cDNA was generated with PrimeScript RT Master Mix (TaKaRa; RR036A). Quantitative PCR was conducted using SYBR Green PCR Master Mix (Vazyme; Q711). Relative gene expression levels were calculated using the 2‐ΔΔCt method, and transcript levels were normalized to the expression of β‐ACTIN. The primers used for qRT‐PCR were as follows:

human EOMES‐F, 5′‐AAATGGGTGACCTGTGGCAAAGC‐3′;
human EOMES‐R, 5′‐CTCCTGTCTCATCCAGTGGGAA‐3′;
human GZMA‐F, 5′‐CCACACGCGAAGGTGACCTTAA‐3′;
human GZMA‐R, 5′‐CCTGCAACTTGGCACATGGTTC‐3′;
human β‐ACTIN‐F, 5′‐CACCATTGGCAATGAGCGGTTC‐3′;
human β‐ACTIN‐R, 5′‐AGGTCTTTGCGGATGTCCACGT‐3′;

### 4.9 Immunoblotting Assay

Cells were lysed with 1% Triton X‐100 lysis buffer (Beyotime; ST795) supplemented with phenylmethylsulfonyl fluoride (Beyotime; ST505), and protease/phosphatase inhibitor cocktail (Thermo Scientific; 78430), and then boiled in 5×SDS loading buffer (Epizyme; LT101) for 5 min. Each sample was loaded on 10% Bis–Tris gels, transferred to PVDF membranes and immunoblotted with the indicated antibodies targeting NEUROD1 (CST; 62953), ICOSLG (Abcam; AB209262), and β‐ACTIN (ABclonal; AC004). The immunoblots were visualized using a chemiluminescence imaging system (Tanon).

### 4.10 Coculture

H82 cells were treated with Prezalumab (MCE; HY‐P99414; 100 nM, 48 h) to block the ICOSLG binding site. A Transwell system (absin; abs7268) was used for coculture of Jurkat cells and H82 cells, with 1 × 10^8^ H82 cells in the upper chamber and 2 × 10^8^ Jurkat cells in the lower chamber. After 24 h of coculture, Jurkat cells in the lower chamber were collected for qRT‐PCR analysis.

### 4.11 Statistical Analysis

Kaplan–Meier analysis and the log‐rank test were used to compare survival outcomes. Multivariate analyses were conducted by Cox proportional hazards regression model. Pearson's chi‐squared test was applied to compare categorical variables. The Mann–Whitney *U* test was utilized for comparisons between two independent samples, while the Kruskal–Wallis test was employed for analysis involving multiple independent samples. Detailed statistical tests were described in the corresponding figure legends. *p* < 0.05 was considered statistically significant. All analyses were conducted using IBM SPSS Statistics v20.0 and R 4.0.5 software.

## Author Contributions

Q. Guo, Y. Chen, and J. Sun for acquisition of data, analysis and interpretation of data, statistical analysis, and drafting of the manuscript. J. Zhang, H. Hu, H. Yu, H. Zhang, and S. Ji for technical and material support. J. Zhang, P. Zhang, H. Hu, and L. Zhang for study concept and design, analysis and interpretation of data, drafting of the manuscript, obtained funding, and study supervision. All authors read and approved the final manuscript.

## Funding

This research was supported by the National Natural Science Foundation of China (Grant No.82473368), the Shanghai Rising‐Star Program (Grant No.24QA2707200), the Shanghai “Rising Stars of Medical Talent” Youth Development Program Youth Medical Talents—Specialist Program (SHWSRS(2025)_071), the Shanghai Science and Technology Committee (Grant No.24SF1904500), Shanghai Municipal Health Commission (Grant No.202540154), Innovation Program of Shanghai Municipal Education Commission (Grant No. 2023ZKZD33), and foundation of Shanghai Pulmonary Hospital (Grant No. TJ‐FK‐YXJC003, LYRC202402, FKLY20004, FKZR2439, Fkyq2509). All these study sponsors have no roles in the study design, collection, analysis, and interpretation of data.

## Ethics Statement

All procedures involving human samples were approved by the Ethics Committee of Shanghai Pulmonary Hospital (Project number: K23‐309) and performed in accordance with the ethical standards.

## Conflicts of Interest

The authors declare no conflicts of interest.

## Supporting information




**Supporting File 1**: mco270782‐sup‐0001‐Figures.docx

**Supporting File 2**: mco270782‐sup‐0002‐Tables.xlsx

## Data Availability

Previously published datasets used during this study are available as follows: sample annotation, processed, and normalized data files of TU‐SCLC cohort were provided in Table S1 in published work, transcriptomic data for IMpower133 patients was via European Genome‐phenome Archive (EGAS50000000138), proteomic and clinical data from Wang et al. cohort was via iProX database (iProX: IPX0004230000). For raw data from the TU‐SCLC cohort, the WES and RNA‐seq raw data have been deposited in the Genome Sequence Archive. The proteomic data have been deposited in the OMIX database, China National Center for Bioinformation/Beijing Institute of Genomics, Chinese Academy of Sciences (Accession no. OMIX002489, https://ngdc.cncb.ac.cn/omix). Other data are available from the corresponding author Dr. Zhang, on reasonable request.
