# Supplementary material for: Inducible T Cell Costimulator Ligand and Inducible T Cell Costimulator Stratification Identify Dichotomous Tumor Microenvironment and Guide Chemo‐Immunotherapy in Small Cell Lung Cancer
Source: MedComm (2020). 2026 May 28;7(6):e70782. doi: 10.1002/mco2.70782 (PMC13239265; doi:10.1002/mco2.70782)
Supplement: Supplementary file 1 — Supporting File 1: mco270782‐sup‐0001‐Figures.docx [file MCO2-7-e70782-s002.docx]

**Inducible T cell costimulator ligand and Inducible T cell costimulator stratification identify** **dichotomous tumor microenvironment and guide chemo-immunotherapy in small cell lung cancer.**

Qiji Guo^1,#^, Yan Chen^1,#^, Jijun Sun^1,#^, Hongyi Zhang^1^, Shuyu Ji^1^, Huansha Yu^2^, Lele Zhang^2,^**^*^**, Haiyang Hu^2,^**^*^**, Peng Zhang^1,^**^*^**, Jing Zhang^1,^**^*^**

^1^Department of Thoracic Surgery, Shanghai Pulmonary Hospital, School of Medicine, Tongji University, Shanghai 200433, China

^2^Central Laboratory, Innovation and Incubation Center, Shanghai Pulmonary Hospital, School of Medicine, Tongji University, Shanghai, 200433, China

^#^These authors contributed equally to this work.

**^*^Correspondence:**

Jing Zhang

Department of Thoracic Surgery, Shanghai Pulmonary Hospital, School of Medicine, Tongji University, Shanghai 200433, China

Email: [meadow@tongji.edu.cn](mailto:meadow@tongji.edu.cn)

Peng Zhang

Department of Thoracic Surgery, Shanghai Pulmonary Hospital, School of Medicine, Tongji University, Shanghai 200433, China

Email: [zhangpeng1121@tongji.edu.cn](mailto:zhangpeng1121@tongji.edu.cn)

Haiyang Hu

Central Laboratory, Innovation and Incubation Center, Shanghai Pulmonary Hospital, School of Medicine, Tongji University, Shanghai, 200433, China

Email: [haiyanghu@cpu.edu.cn](mailto:%20haiyanghu@cpu.edu.cn%20n)

Lele Zhang

Central Laboratory, Innovation and Incubation Center, Shanghai Pulmonary Hospital, School of Medicine, Tongji University, Shanghai 200433, China.

Email: [zhanglele@tongji.edu.cn](mailto:%20haiyanghu@cpu.edu.cn%20n)

**Supplementary Figures**


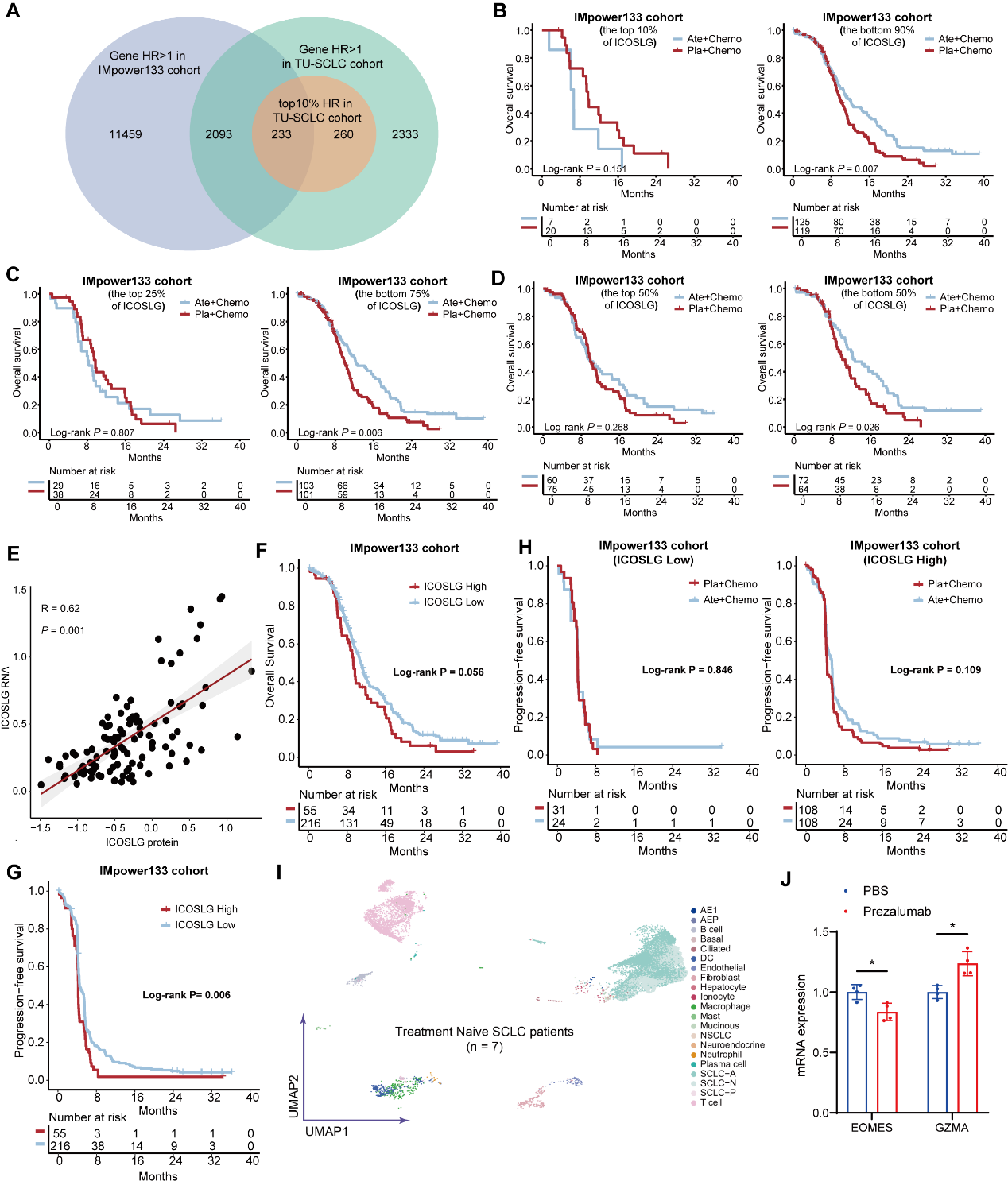


**Figure S1. Characteristics of ICOSLG. (A)** Venn plot showing the exploration of factors associated with prognosis and efficacy of chemo-immunotherapy in SCLC. **(B-D)** Kaplan-Meier analysis of OS among patients who received chemo-immunotherapy or chemotherapy alone, stratified by ICOSLG cut-off, in the IMpower133 cohort. **(E)** The Spearman correlation coefficient between RNA level and protein level of ICOSLG. **(F-G)** Kaplan-Meier analyses of OS and PFS between the ICOSLG low subgroup and ICOSLG high subgroup in the IMpower133 cohort. **(H)** Kaplan-Meier analyses of PFS between patients treated with chemo-immunotherapy or chemotherapy alone during ICOSLG low subgroup and ICOSLG high subgroup in the IMpower133 cohort. **(I)** UMAP plot of 7 treatment-naive patients from the Rudin *et al.* cohort, color-coded to indicate the major cell lineages. **(J)** Relative mRNA expression levels of the exhaustion marker EOMES and the effector molecule GZMA in Jurkat T cells following co-culture with H82 cells. Prior to co-culture, H82 cells were pre-treated with either a PBS control (blue bars) or an anti-ICOSLG neutralizing antibody (Prezalumab, red bars). Data were presented as mean ± SD, and *p* values were calculated by two-sided Student's t test. *, *P* < 0.05.


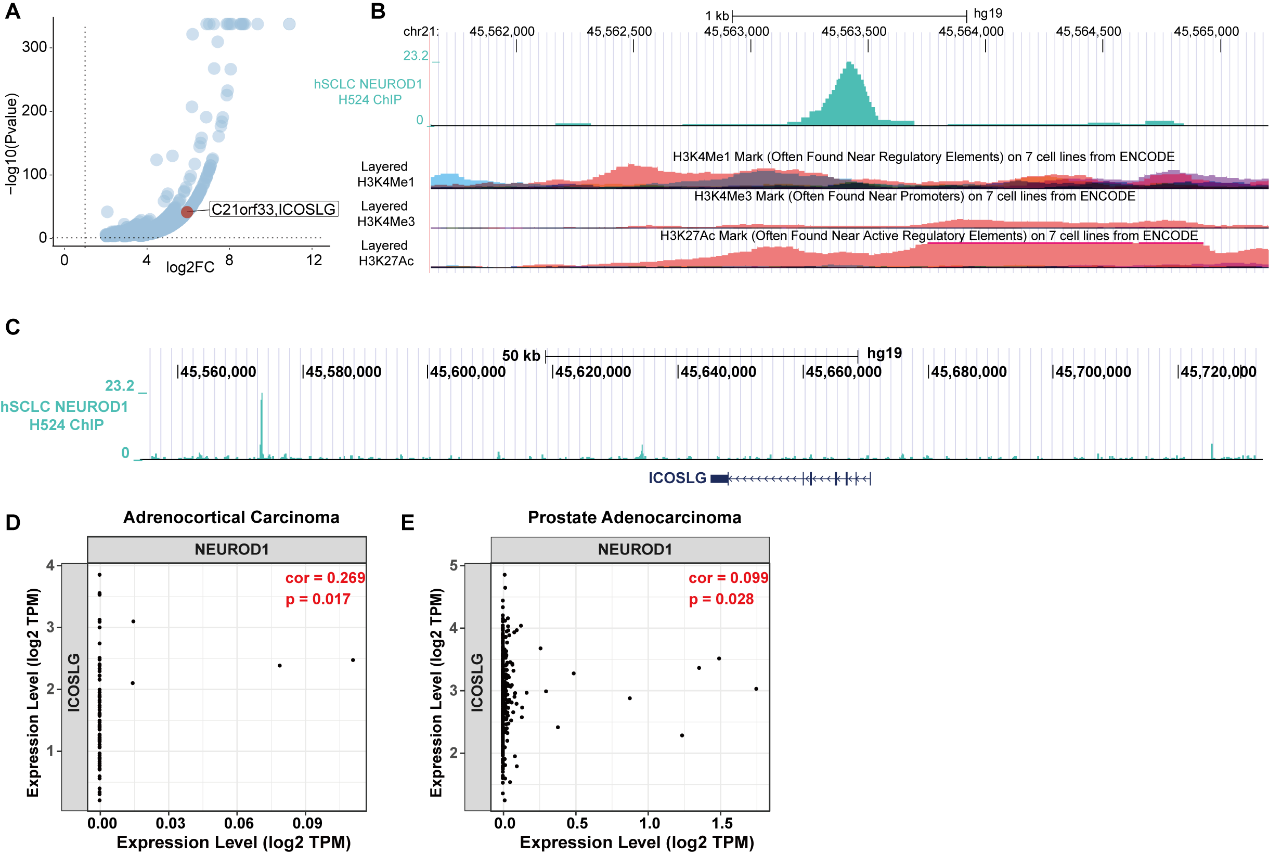


**Figure S2. Potential regulation mechanism of ICOSLG by NEUROD1.** **(A)** Fold increase of peaks of NEUROD1-high cells relative to ASCL1-high cells. A maximum of two target genes were assigned to each peak using GREAT software. **(B)** Histone modification patterns of the peak assigned to ICOSLG. **(C)** Genomic landscape illustrating the relative location of the NEUROD1 binding peak upstream of the ICOSLG gene locus. **(D–E)** Correlation analysis between NEUROD1 and ICOSLG expression levels in Adrenocortical Carcinoma and Prostate Adenocarcinoma. Pearson correlation coefficients and *p*-values are indicated.


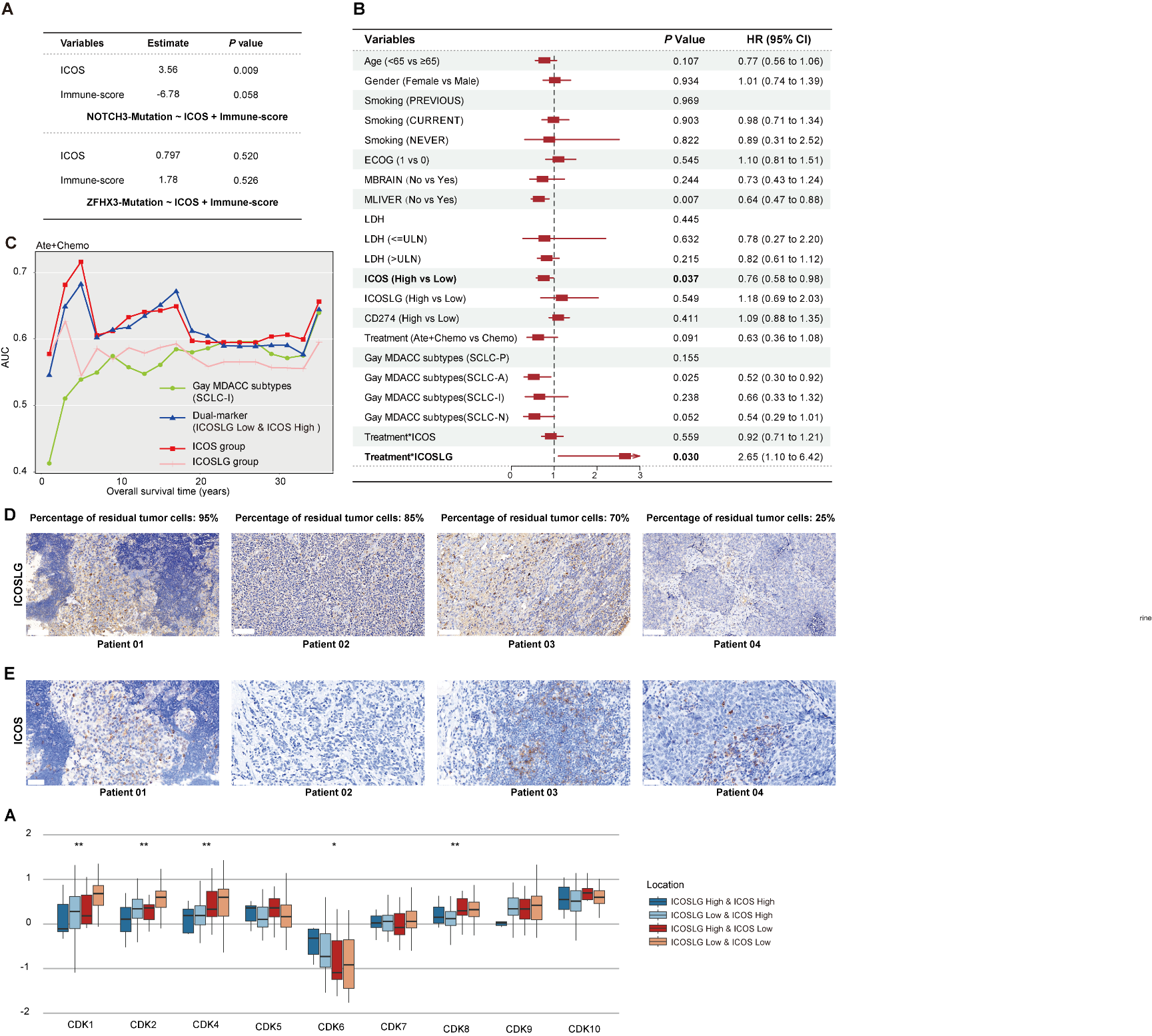


**Figure S3. Combined analysis of ICOS and ICOSLG dual markers.** **(A)** Joint analysis of ICOS with ZFHX3 or NOTCH3 mutations after adjusting for Immunescore. **(B)** Forest plot of multivariate Cox proportional hazards regression analysis including treatment, continuous biomarker value, and their interaction while adjusting for age, gender, ECOG PS, liver or brain metastases, LDH, smoking status, SCLC transcriptional subtype, and PD-L1 expression. **(C)** Time-dependent AUC of dual-marker model versus single markers and versus SCLC-I. **(D-E)** Representative immunohistochemical staining of ICOSLG and ICOS in patient tissue sections.

**
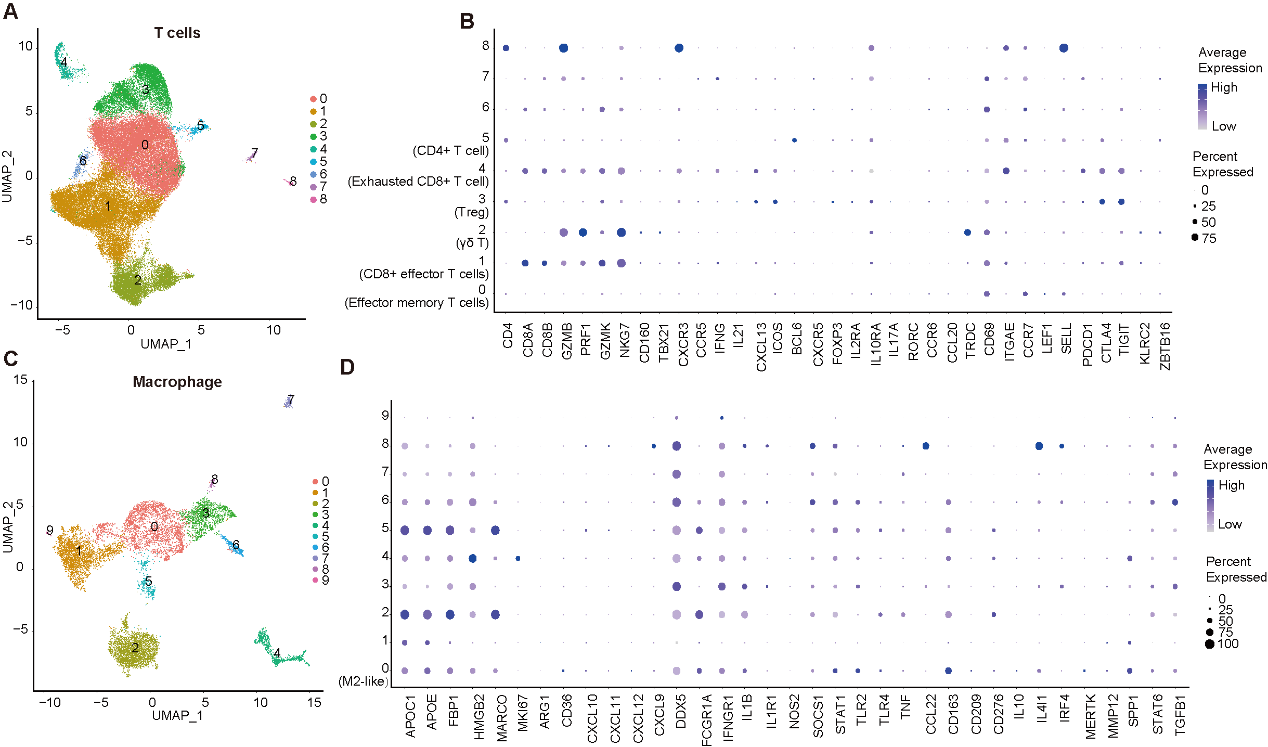
**

**Figure S4. Annotation of T cells and macrophages in SCLC. (A-B)** UMAP plot showing T cells and macrophages colored by different clusters. **(C-D)** Dot plot displaying the average expression and expressed percentage of representative markers of each T cell and macrophage cluster.


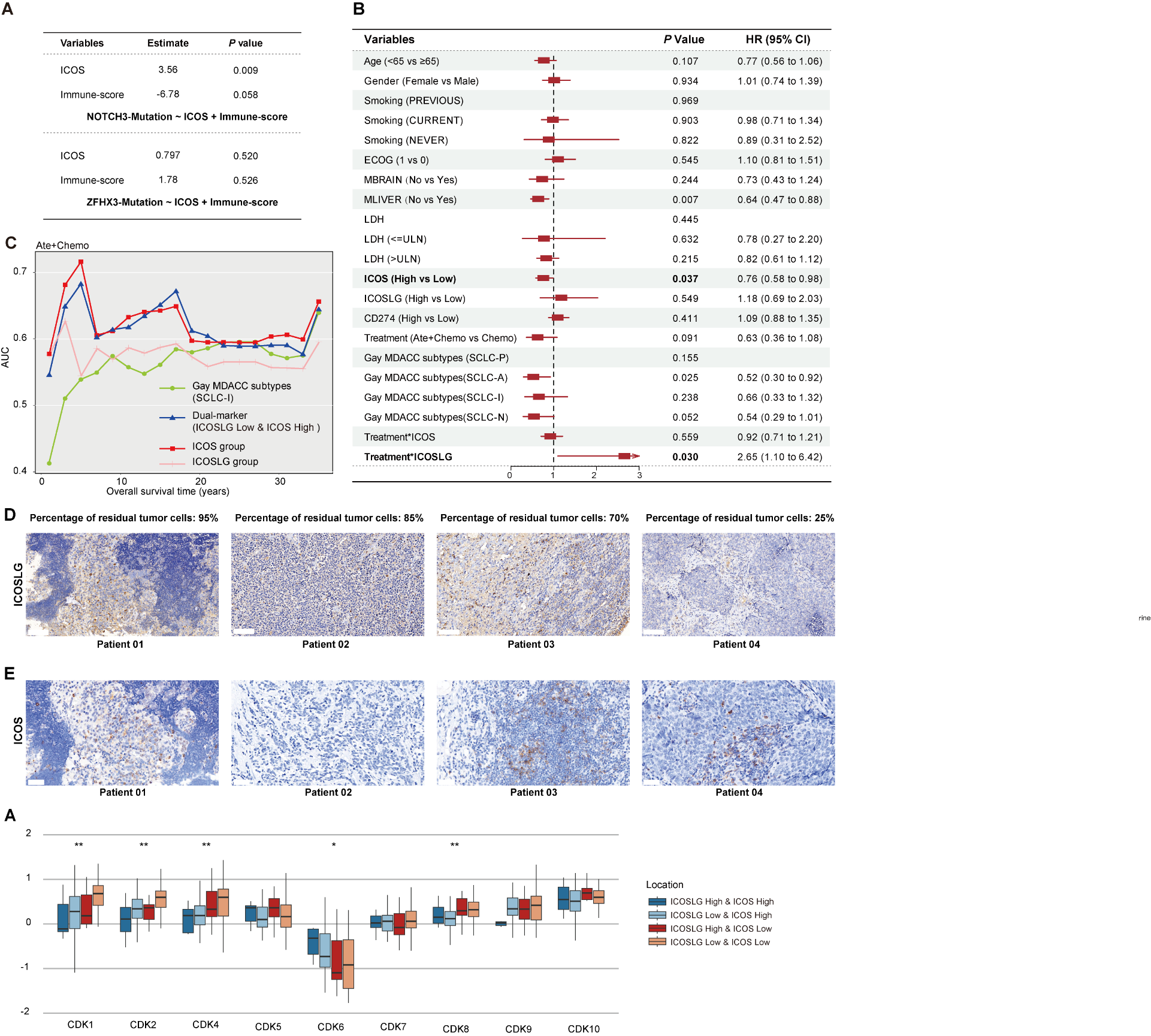


**Figure S5.** The protein level of CDK molecules across four clusters. Statistical significance was determined by the Kruskal-Wallis test. *, *P* < 0.05; **, *P* < 0.01; ***, *P* < 0.001.

**Table S1.** List of candidate genes associated with prognosis and chemo-immunotherapy efficacy identified in IMpower133 and TU-SCLC cohorts.

**Table S2.** Associations between genomic mutations and ICOS expression before and after adjustment for immune infiltration scores.

**Table S3.** List of gene signatures and definitions used for ssGSEA and GSEA analyses.
